# Microprocessor of microRNAs: regulation and potential for therapeutic intervention

**DOI:** 10.1186/1476-4598-9-134

**Published:** 2010-06-01

**Authors:** Kevin J Beezhold, Vince Castranova, Fei Chen

**Affiliations:** 1 Laboratory of Cancer Signaling and Epigenetics, Health Effects Laboratory Division, Pathology and Physiology Research Branch, National Institute for Occupational Safety and Health, 1095 Willowdale Road, Morgantown, WV 26505, USA; 2 Cancer Cell Biology Program, West Virginia University, WV 26506, USA

## Abstract

MicroRNAs (miRNAs) are a class of small, noncoding RNAs critically involved in a wide spectrum of normal and pathological processes of cells or tissues by fine-tuning the signals important for stem cell development, cell differentiation, cell cycle regulation, apoptosis, and transformation. Considerable progress has been made in the past few years in understanding the transcription, biogenesis and functional regulation of miRNAs. Numerous studies have implicated altered expression of miRNAs in human cancers, suggesting that aberrant expression of miRNAs is one of the hallmarks for carcinogenesis. In this review, we briefly discuss most recent discoveries on the regulation of miRNAs at the level of microprocessor-mediated biogenesis of miRNAs.

## Introduction

MicroRNAs (miRNAs) are endogenously synthesized small non-coding RNAs that regulate gene expression by interfering with protein translational machinery and/or inducing degradation of target mRNAs [1]. Since the discovery of miRNAs, much effort has been made to understand the mechanisms by which miRNAs are synthesized and involved in cell lineage development and human diseases, especially, cancer. It is imperative that scientists continue to delineate how the biogenesis of these miRNAs is controlled by the cellular processing machinery, so that one may better understand how to modulate their expression or function as it contributes to a unique disease state. Recent research shows the involvement of additional proteins that modulate the function of the miRNA processing machinery, the Drosha processing complex, or microprocessor. This article reviews these new findings and discusses the potential for targeting these regulatory pathways in cancer therapy.

### 1. MicroRNA biogenesis

**Figure 1:**
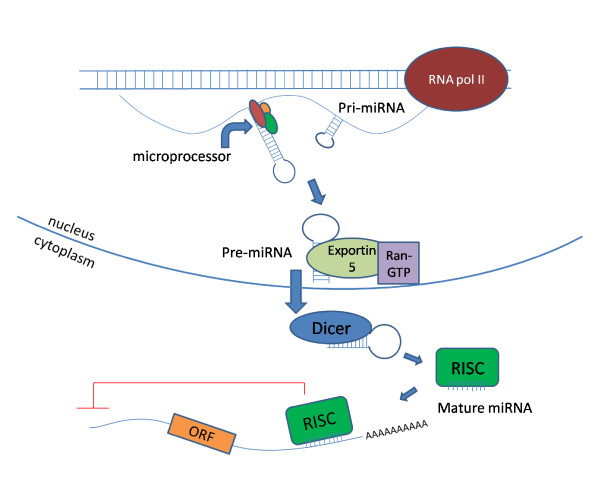
**MicroRNA (miRNA) production and processing**. The pri-miRNA transcript is transcribed by RNA polymerase II. The stem loop structure is cleaved off by the microprocessor to generate pre-miRNA. The pre-miRNA is exported to the cytoplasm by exportin5 in a ran-GTP dependent manner. Once in the cytoplasm, the pre-miRNA is processed by Dicer creating a single stranded mature miRNA. This mature miRNA is bound by the RISC complex, guiding it to the 3'UTR of target mRNAs, leading to repression of protein expression.

It has been well-established that the biogenesis of microRNAs (miRNAs) involves three step-wise processes, including transcription of primary miRNAs (pri-miRNAs) from the miRNA genes [2], partially processed precursor miRNAs (pre-miRNAs) in nuclei [3] and the mature miRNAs that were generated in the cytoplasm (Fig. 1). Pri-miRNA is typically a large RNA polymerase pol II-derived transcript whose tertiary structure forms stem loop structures. The stem loop is cleaved off by the microprocessor machinery, Drosha complex, to form ~60-100 nucleotide long pre-miRNA, which is further processed into ~22 nucleotide long mature miRNAs by Dicer, a RNase III enzyme, following translocation from the nuclei to cytoplasm [4].

After successful cleavage, the pre-miRNA is bound by exportin-5 in a ran-GTP dependant manner and exported from the nucleus [5-7]. Binding of pre-miRNA by exportin-5 is dependent upon the stem of the miRNA, requiring a length of 16-18 base pairs, and alterations in the 3' overhang will affect the efficiency of exportin-5 binding[8]. Interestingly, reduced binding of exportin-5 by reduction of the protein itself or alteration in the miRNA structure causes a reduced expression of the mature miRNA, without buildup of pre-miRNA in the nucleus [5]. This suggests that exportin-5 may play a protective role during miRNA transport to the cytoplasm [8]. Once in the cytoplasm, the pre-miRNA is released from exportin-5 after the hydrolysis of GTP, and is free to be processed further.

In the cytoplasm, pre-miRNA undergoes the next step of processing mediated by Dicer to produce the mature miRNA. The RNase III enzyme, Dicer, was found to cleave RNAs into ~22 nucleotide products [9-11]. This cleavage occurs in an ATP independent manner, through which the loop structure and 3' overhang are removed [12]. Recognition and correct cleavage of the pre-miRNA are determined by the different domains of Dicer. Dicer contains a PAZ domain which recognizes the 3' end of the pre-miRNA, and the rest of the molecule acts as a molecular ruler directing the RNase III domains to cleave the 3' overhang and the loop structure to generate the mature miRNA [13]. After cleavage, one strand of the miRNA duplex is preferentially incorporated into the RISC complex. The selection of one strand over the other is based upon thermodynamic properties of the duplex, and the strand with the less thermodynamical stability at the 5' end is usually selected [14]. The mature miRNA bound to RISC then associates with an Argonaute protein, most commonly Ago2, and directs binding of the RISC complex to partially complementary sites in the 3'-UTRs of targeting mRNAs [15].

Previous observations suggested that the specificity of miRNA is determined by the sequence complementarity between bases 2-8 on the 5' end of the miRNA, termed the seed sequence, and the target mRNAs [16,17]. A recent study appears to oppose this seed sequence pairing mechanism, and identifies binding and repression of mRNA by several "seedless" miRNA-mRNA duplexes [18]. In a microarray study for proteins down regulated by miR-24 expression, it was observed that multiple genes whose expression was reduced do not have predictable target sequences. Using an algorithm that does not require a seed match, it was further confirmed that the miR-24 targeting sequences are indeed within the 3' UTRs of the repressed genes [18].

There are multiple mechanisms by which miRNAs downregulate gene expression, some of which are still in controversy. These mechanisms have been reviewed in-depth elsewhere [19]. Briefly, mRNAs have been observed to be repressed by three major processes including endonucleolytic cleavage, mRNA degradation by deadenylation, and inhibition of translation initiation. Similar to siRNA-mediated mRNA degradation, the endonucleolytic cleavage of mRNA by miRNA requires perfect or near perfect complementarity between miRNA and the target mRNA. If such a condition is satisfied, proteins within the RISC complex are then able to cleave the mRNA, leading to its degradation and silencing [20]. This gene silencing process has been shown to occur in multiple organisms including mammals. Of note however, this mechanism of gene regulation rarely occurs in mammalian cells because nearly all miRNA-mRNA interactions have significant mismatches [17,21-23].

Inhibition of translation initiation is another widely studied mechanism of miRNA-induced gene silencing. In 2005 Pillai et al. found that a miRNA-targeted reporter mRNA sedimented with small polysomes in HeLa cells, which indicates the repression occurred at translation initiation. In the same publication, they also showed that reporter constructs which are not dependant on the 7-methyl guanosine cap structure did not undergo repression, and suggested that miRNPs are capable of binding to the cap structure [24]. A motif was later found within AGO2 that was indeed capable of binding to the 5' cap structure of mRNA, which could then compete with eIF4E that is necessary for initiation of translation [25].

Cellular localization is another mechanism by which miRNA might mediate repression of mRNA translation. There is evidence suggesting that some miRNP-bound mRNAs localize to p-bodies within the cytoplasm. The p-bodies are cytoplasmic foci that contain mRNAs that are not actively undergoing translation. The p-bodies also contain proteins that are responsible for mRNA degradation [26]. Several observations suggest that mRNAs within the p-bodies are being repressed by miRNA that are co-localized in these foci. However, a detailed mechanism by which the repression occurs has yet to be fully determined. In addition, the deadenylation of mature mRNA has been implicated as a mechanism by which miRNAs are able to repress protein translation within the p-bodies. It was believed that GW182, a protein important for deadenylation of mRNAs, localizes in p-bodies and is able to interact with AGO1. This interaction leads to recruitment of decapping complexes and subsequent degradation of the miRNP-bound mRNA [27-29].

### 2. Microprocessor and pri-miRNA processing

The pri-miRNAs transcribed from miRNA genes usually exhibit a size of several thousands of nucleotides long, whereas the size of mature miRNAs is only about 22 base pairs [30]. As briefly mentioned earlier, the generation of mature miRNA, encoded by either an intron of protein-coding gene or intergenic non-coding transcription unit, requires two sequential endonucleolytic cleavages by RNase III enzymes. A nuclear protein, Drosha, is the first enzyme to catalyze such processing. It is believed that Drosha is able to cleave intronic pri-miRNA without interference with the splicing of the precursor mRNA (pre-mRNA) [31]. There is also evidence indicating that cleavage of pri-miRNA by Drosha can be closely coupled with transcription of the pri-miRNAs from either intronic or intergenic miRNA genes [31].

**Figure 2:**
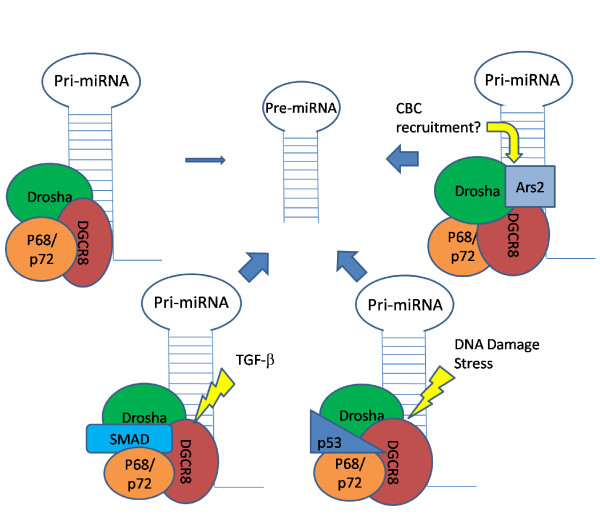
**Modulation of microprocessor function by SMAD, p53 and Ars2 in response to TGF-β, p53 and stress signaling, respectively**.

Accumulating evidence suggests that processing of pri-miRNA by Drosha itself is insufficient and often erroneous, since imprecise cleavage occurred by the recombinant Drosha protein [32]. Inaccuracy of pri-miRNA cleavage will result in production of pre-miRNAs with altered hairpin secondary structure and identities. To ensure efficient and precise processing of pri-miRNA by Drosha, a number of co-factors are necessarily needed. Indeed, protein fractionation by affinity chromatography revealed that Drosha is present in protein complexes with different sizes in vivo [32,33]. The study performed by Gregory et al. showed association of Drosha with 2 different complexes, a large complex with ~20 proteins and a small complex consisting of Drosha and DiGeorge syndrome critical region 8 (DGCR8) [32]. The association of Drosha with these proteins forms microprocessors to ensure the fidelity and activity of Drosha cleavage on pri-miRNA [32]. DGCR8 is a protein found within the DiGeorge syndrome critical region and was revealed to be essential for the processing of pri-miRNA to pre-miRNA by Drosha. At about the same time, Han et al. also identified DGCR8 as a critical player in miRNA processing and demonstrated that the microprocessor complex may be composed of multiple DGCR8 and Drosha molecules, possibly creating a dynamic processing structure [33]. Because most pri-miRNAs have similar structures typified by a terminal loop, a double stranded stem, and single stranded flanking sequences, it was speculated that this common structure may be the determining factor that regulates pri-miRNA cleavage (Fig. 2). There is evidence indicating that both the terminal loop and single stranded flanking sequences are important for processing efficiency [4,34,35]. An in-depth look at the molecular mechanisms controlling the binding of pri-miRNAs by the microprocessor complex suggests that DGCR8 is responsible for the binding of the complex to the pri-miRNA stem-loop. DGCR8 recognizes both the single stranded flanks and the double stranded stem, and then acts as a ruler guiding Drosha to cleave the molecule in the correct place, 11 base pairs up the stem from the ssRNA-dsRNA junction (Fig. 2) [36].

In addition to DGCR8, other well-characterized proteins in the microprocessor which facilitate the activity of Drosha include two DEAD-box RNA helicases p68 and p72 [32,37]. Both p68 and p72 are originally identified as ATP-dependent RNA helicases important for pre-mRNA and pre-rRNA splicing by association with spliceosome complexes [38]. Genetic disruption of either p68 or p72 in mice is lethal.

Surveying miRNA expression profiling using embryo fibroblast cells (MEF) suggests about 35% reduction of pre-miRNA and mature miRNA expression in p68 or p72 gene knockout MEFs relative to the wild type MEFs. Intriguingly, the level of pri-miRNAs is comparable between the knockout MEFs and the wild-type MEFs, indicating contribution of p68 or p72 to the Drosha-mediated pri-miRNA processing [39]. Direct evidence of p68 and p72 in pri-miRNA processing was provided by demonstrating their interaction with the Drosha and DGCR8 proteins in an immunoprecipitation-mass spectromic analysis [32,37].

### 3. Regulation of the microprocessor by SMADS

The first evidence showing additional proteins binding to and modulating the function of the Drosha complex was provided by Davis et al. in 2008 [40]. Through studying which miRNAs might play a role in the phenotypic changes of the vascular smooth muscle cells in response to TGF-β signaling, they found that miR-21 and miR-199a were induced by BMP4 and TGF-β stimulation. Further studies indicate that such an induction occurred at the post-transcriptional level because BMP4 or TGF-β rapidly induces pre-miR-21 and mature miR-21 but not pri-miR-21. Indeed, the expression of pri-miR-21 stayed stable following the activation of the TGF-β signaling. The induction of pre-miR-21 and miR-21 by BMP4 or TGF-β was blocked in the cells when expression of SMAD proteins was repressed by siRNA knock-down. Previous studies suggest that the MH2 domain on the carboxyl-terminus of SMAD proteins is capable of binding to p68, the RNA helicase associated with the Drosha complex [41]. This conclusion was supported by Davis et al. [40] who demonstrated direct interaction of p68 with SMAD1, 3, and 5 in a GST-pull down experiment. The interaction of Drosha with the p68/SMAD complex occurred only under conditions where the miRNA transcripts were intact. Furthermore, RNA co-immunoprecipitation confirmed the specific regulation of SMADs on the microprocessors of miR-21 and miR-199a, but not miR-214, in response to BMP4 or TGF-β (Fig. 2).

Activation of SMAD proteins by TGF-β has long been assumed as a mechanism for inducing epithelial to mesenchymal transition (EMT) and overall cancer cell growth. Adding to this role, the above study demonstrates an additional function of TGF-β being able to increase the expression of miRNAs 21 and 199a. MiR-21 is the most commonly over-expressed miRNA in cancers [42]. Over-expression of miR-21 has been reported in more than 15 different malignancies [43]. The oncogenic potential of miR-21 is largely attributed to its involvement in several intracellular signaling pathways, including the activation of AKT [44] and antagonizing the expression of the pro-apoptotic protein PDCD4 [45]. Expression of miR-199a has also been associated with cancers. One study suggests that leukemias with higher expression of miR-199a exhibit a worse prognosis [46]. Several other studies, however, show that a loss in expression of miR-199a, enhanced tumor progression by an enhancement of IKKβ expression and its induced inflammatory and tumorigenic signals in ovarian cancer [47].

TGF-β and SMAD4 have also been recognized as key players in inducing transcription of miR-155 and miR-214, two other oncogenic miRNAs [48]. Elevated miR-155 represses RhoA protein expression, reduces the ability of epithelial cells to form tight junctions, and enhances a tumors' ability of metastasis [48]. In ovarian cancer, miR-214 is overexpressed, leading to a sustained activation of the Akt kinases by down-regulation of PTEN, a negative regulator of Akt signaling. Decrease in PTEN expression, thus, causes an aberrant activation of Akt and resistance of the tumor cells to chemotherapy, such as cisplatin treatment [49].

The above reports detailing regulation of Drosha processing by SMADs in response to TGF-β signaling are of particular interest. This modulation appears to be specific to a subset of miRNAs, and it will be important to determine what other miRNAs are regulated by this same mechanism and what molecular events govern such regulation. It appears that the binding of SMAD proteins to the Drosha microprocessor stabilizes the formation of the complex on a specific set of pri-miRNAs. Accordingly, it will be important to determine the downstream targets of these miRNAs and whether regulation of miRNA processing is a major mechanism of TGF-β in cell transformation and carcinogenesis.

### 4. p53 and miRNA processing

The tumor suppressor p53 is perhaps the most intensively studied protein in cell biology and cancer. As a transcription factor, the tumor suppressor function of p53 is achieved largely by transcriptional up-regulation of a number of pro-apoptotic proteins. A recent study by Suzuki and colleagues demonstrates a novel mechanism of p53 in tumor suppression by regulating miRNA biogenesis at the level of Drosha microprocessor [50]. The interaction of p53 with p68, a protein associated with the Drosha microprocessor, has been previously demonstrated [51]. In the latest study, it was noted that p53 is capable of interacting with p68 and p72, both of which are Drosha-associated RNA helicases (Fig. 2). Using doxorubicin as a DNA damaging agent and p53 inducer in human colon cancer cell line HCT116, the expression of a subset of miRNAs was up-regulated. These miRNAs include miR-15a, miR-16-1, miR-23a, miR-26a, miR-103, miR-143, miR-145, miR-203, as well as miR-34a that had previously been determined to be induced by p53 [52]. Upon examination of the expression levels of pri-, pre- and mature miRNAs for each of the miRNAs regulated by doxorubicin, as expected, all species of miR-34a, a transcriptional target of p53, were upregulated. Interestingly, several other miRNAs showed increases of pre- and mature miRNA species, but not the pri-miRNA transcripts, suggesting that the regulation of p53 for some of these miRNAs is independent of transcription. Furthermore, cancer-associated p53 mutants fail to bind p68 or induce miRNA processing. Overexpression of each of these p53-regulated miRNAs substantially decreased the rate of cell proliferation. Taken together, these data clearly indicate that mutation of p53 in cancers hinders the maturation of several miRNAs important for tumor suppression and leads to an increased tumorigenic potential.

The involvement of p53 in miRNA biogenesis post-transcriptionally provides evidence of a global control mechanism for subsets of miRNAs involved in similar cellular functions. The p53 transcriptional target, miR-34a, was shown to repress tumor progression in multiple cancers [53-55] and has recently been found to target c-met and Notch1/Notch2 in glioblastomas [56]. Interestingly, expression of miR-15a and miR-16-1 is often reduced in tumors. Both miR-15a and miR-16-1 have been shown to target the anti-apoptotic protein, BCL2 [57,58]. This cluster of miRNAs is also observed to be able to target additional proteins such as Cyclin D1 and WNT3A, which promote tumorigenesis [59]. Decreased expression of miR-143 and miR-145 has been strongly linked to colon cancer [60-62]. MiR-143 has been shown to target KRAS [63] and DNA methyltransferase 3A [62]. In breast cancer, loss of miR-145 resulted in an elevation of rhotekin (RTKN), a scaffolding protein for Rho-GTP that is involved in cell proliferation [64].

The involvement of p53 in miRNA biogenesis was unexpected. The capability of p53 to regulate miRNA expression obviously strengthens its tumor suppressor function further. The questions that remain unanswered are why association of p53 with the Drosha microprocessor only regulates a selected subset of miRNAs but not others and how does p53 alter the recognition and processing dynamics of the Drosha complex toward the pri-miRNA repertoire. Nevertheless, the discovery of p53 regulation on miRNA processing confirms that p53 is a powerful suppressor for cancer formation. Loss of p53 as observed in multiple cancers, therefore, will not only weaken the checkpoint mechanisms of the cells but also impair the generation of those tumor suppressor-like miRNAs.

### 5. ARS2 regulation of miRNA processing

Arsenic Resistance protein 2 (Ars2) is a protein whose expression is strongly linked to the proliferation of cells especially during embryonic development [65]. Recently two studies have been published linking Ars2 expression to miRNA processing. The study by Gruber et al. [66] show that in addition to its role in cell proliferation, depletion of Ars2 in mammalian cells repressed miRNA-mediated silencing of reporter genes. After siRNA knockdown of Ars2, the ability of let-7 to repress the expression of a luciferase reporter construct was significantly reduced. Addition of the let-7 duplex RNA was able to reverse the loss of reporter repression, indicating that Ars2 does not function down-stream of Drosha processing. Immunoprecipitation of Drosha proteins was able to pull down Ars2, while the same experiment with Dicer did not. This further indicates that repression of miRNA function occurs at the Drosha processing step (Fig. 2). Previous research performed on SERRATE, a plant homolog of Ars2, indicates that Ars2 was responsible for regulating the appropriate processing of miRNA by Drosha [67]. However, Gruber et al. found that Ars2 was not required for processing of all miRNAs, but only for a subset containing let-7 and miR-21. After screening for factors important in antiviral defense in *Drosophila*, Sabin et al. simultaneously observed a similar activity of Ars2 in miRNA processing in *Drosophila *with viral infection [68]. They identified that Ars2 is critical for intrinsic antiviral defense in *Drosophila*. Loss of Ars2 leads to a pronounced increase in viral replication of several RNA viruses in both cell culture and adult flies. Using an over-expression strategy, Sabin et al. [68] also demonstrate that Ars2 was capable of binding to Pasha, also known as DGCR8 in mammals, the double-stranded RNA binding partner of Drosha. Additional experiments implied interaction between Ars2 and the nuclear cap-binding complex (CBC) that recognizes and binds to the 5'-cap of pri-miRNA transcripts. Based on all of these observations, two nonexclusive models for the role of Ars2 in miRNA processing were proposed. The first is the so-called bridging model, in which both Ars2 and CBC bind pri-miRNA transcripts followed by recruitment of the Drosha microprocessor by Ars2. In the second model, Ars2 acts as a cofactor for Drosha's enzymatic activity by enhancing the overall processing activity and fidelity of the microprocessor [68].

The discovery of Ars2 in miRNA processing further emphasizes the point that miRNA biogenesis and activity are highly regulated processes involving multiple proteins at various stages. Although Ars2 is a protein which has not been extensively studied, early reports indicating its contributions to cell proliferation and more recent studies showing its role in miRNA processing suggest that Ars2 may be a potential target for therapeutic intervention in various disease states including cancer.

### 6. Cell-to-cell contact affects miRNA processing

The regulation of microRNA processing has been found to be affected by the confluence of the culture or the intensity of cell-to-cell contacts. Hwang et al. found that as cultures reach confluence, the expression of most of the miRNAs that they studied also increased [69]. They were able to show that this effect occurred across multiple cell lines and was typified by an accumulation of pre- and mature miRNA. This change in miRNA levels appears to be independent of the status of cell proliferation, conditions of the cell culture media, or the density of the cells in culture. This indicates that an increase in cell-to-cell contacts was the impetus for the increase in miRNA processing. The authors further determined that the abundance of pri-miRNA transcript was not increased by the status of cell confluence with the interesting exception of miR-34a. Such information along with additional experiments indicated that this regulation was caused by an increase in efficiency of the Drosha microprocessor as well as formation of mature miRISC complexes at the Dicer processing step [69].

In light of the study discussed earlier indicating the role of p53 in Drosha processing, it is very likely that this regulation could be due to p53 activity. Along with the non-transcriptional induction of miR-15a, miR-16, miR-26a, and miR-145, the observation that miR-34a was the only transcriptionally regulated miRNA in both studies is the key to linking these two studies together. Furthermore, p53 has been implicated as an important mediator for the density-dependant growth inhibition of cells. One study shows that inhibition of p53 led to the loss of density-dependent growth inhibition, leading to increased cell density and decreased apoptosis. While no increase of p53 expression was observed, basal levels were sufficient to cause growth arrest [70]. The studies by Suzuki et al. and Hwang et al. may, at least in part, be able to explain how p53 can cause density-dependent growth inhibition. While most of the miRNAs observed to be up regulated by Hwang et al. are involved in growth inhibition, some are strongly associated with proliferation and tumorigenesis, like miR-21. This may indicate that additional proteins are involved in the effect of cell confluence- or cell-to-cell interaction-mediated miRNA processing. It would be of interest to determine if the effect seen on processing efficiency by Dicer and miRISC formation is also due to p53 activity or to additional modulators of miRNA processing.

### 7. Inhibition of miRNA biogenesis by estrogen

Estrogen hormones are well known regulators for transcription and post-transcriptional events of a number of genes through binding to their specific nuclear estrogen receptors (ERs), ERα or ERβ. Although both receptors exhibit a similar affinity toward estrogen, a distinction in tissue distribution between ERα and ERβ has long been recognized. ERα is mainly found in endometrium, breast cancer cells, ovarian stroma cells, and in the hypothalamus, whereas ERβ appears to be ubiquitously expressed. By using embryos derived from female mice with a genetic deficiency of ERα, a recent study by Yamagata et al. [71] reported an upregulation of some miRNAs. Conversely, such an upregulation was reversed by estrogen (E2) treatment, suggesting that estrogen and its receptor signaling are negative regulators for certain miRNAs, including miR-16, miR-26a, miR-29a, miR-125a, miR-143, miR-145, miR-195, etc.. Further studies indicate that the negative regulation of ERα on these miRNAs occurred at the level of pri-miRNA processing, rather than transcription. A direct physical association of the E2-bound ERα with Drosha microprocessor components, p68 and p72, was noted in an immunoprecipitation assay. The regulation of ERα on miRNA biogenesis was also validated in human cells. Collectively, these data suggest that E2-ERa signaling is antagonistic for miRNA processing, possibly through direct interaction between ERα and p68/p72, leading to dissociation of the Drosha microprocessor from a subset of pri-miRNAs.

The evidence showing inhibitory roles of ERα on the biogenesis of a select subset of miRNAs provides a new explanation for the molecular mechanisms of the ERα positive breast cancers. Due to its negative regulation on some of these tumor suppressor-like miRNAs, including miR-16 and miR-26a, ERα can amplify the tumorigenic signals from VEGF [71], EZH2 [72] and some oncogenes that are targeted by miR-16 or miR-26a in breast epithelial cells. Thus, there is strong indication for pursuing an ERα-based therapeutic approach. First, suppression of the ERα signaling by selective ERα modulators, such as tamoxifen, can inactivate transcriptional regulation of ERα on some growth factors important for the transformation of the cells. Second, blocking ERα signaling will enhance the tumor suppressive potential of the cells by promoting the biogenesis of those tumor suppressor-like miRNAs, which limit the growth and vascularization of the tumors.

## Summary and Conclusions

An underlying theme in the regulation of miRNA biogenesis at the Drosha processing step seems to be that regulatory proteins selectively alter the expression of certain subsets of miRNAs. This is not entirely surprising, as the cellular functions of miRNAs are diverse, and global up-regulation or down-regulation of all miRNAs might cause havoc on cellular systems. The miRNA subsets whose expression are altered by the regulatory proteins discussed above seem to be in line with the traditionally accepted roles for those proteins. As more and more regulatory proteins are discovered for all steps of miRNA production and processing, it is most likely that this theme will be extended. The activation of Smad proteins is associated with cell growth and transformation, and the miRNAs that are regulated by Smads are associated with similar effects. On the other hand, expression and activation of p53 is a well known mechanism of cell cycle arrest and apoptosis, and its downstream miRNAs can be effectors of the same pathways. While Ars2 is not a very well-studied protein and its detailed function remains to be fully elucidated, recent research shows that it is involved in cell proliferation. Likewise, the miRNAs that Ars2 has been shown to regulate are involved in similar cellular functions. Additional research should be done to delineate exactly which miRNAs are in each regulated subset. This information would be useful for determining if therapeutic intervention of proteins, such as Ars2, would be a fruitful endeavor.

One exception to the theme is the observed activation of miRNA biogenesis by cell-to-cell contacts. This effect appears to be global (with few exceptions), including the expression of miRNA that have historically opposite effects. Cellular signaling that occurs during quiescence is highly complex, and the study by Hwang et al. now places miRNA expression into the mix [69].

As the study of miRNA biogenesis continues, it is apparent that more proteins will be discovered to play regulatory roles at various processing steps. It will be important to determine whether or not these proteins or processing pathways are legitimate targets for therapeutic intervention. It is becoming increasingly clear that subsets of miRNA play important roles in multiple disease states. If there are master regulators of expression of these subsets, they could be potential targets for intervention and may be critical for the alleviation of symptoms or reversal of disease.

## Competing interests

The authors declare that they have no competing interests.

## Authors' contributions

KJB drafted the first version of the manuscript and performed the major experiments on miRNA; VC participated in the design of the study and provided suggestions of manuscript; FC conceived of the study, completed the final version of the manuscript and approved the submission of the manuscript. All authors read and approved the final manuscript.
